# Reliability and validity of the original and brief German version of the Maternal Antenatal Attachment Scale (MAAS): Longitudinal study findings

**DOI:** 10.1371/journal.pone.0316374

**Published:** 2024-12-31

**Authors:** Franziska Lehnig, Katja Linde, Viktoria Schmidt, Michaela Nagl, Julia Martini, Holger Stepan, Anette Kersting

**Affiliations:** 1 IFB AdiposityDiseases, Leipzig University Medical Center, Leipzig, Germany; 2 Department of Psychosomatic Medicine and Psychotherapy, University of Leipzig, Leipzig, Germany; 3 Faculty of Medicine, Department of Psychiatry and Psychotherapy, Carl Gustav Carus University Hospital, Technische Universität Dresden, Dresden, Germany; 4 Department of Obstetrics, University of Leipzig, Leipzig, Germany; Universitatsklinikum Schleswig Holstein Campus Lubeck, GERMANY

## Abstract

**Background:**

Maternal-foetal attachment (MFA) seems essential for adapting to motherhood and the healthy development of the child, with direct implications for clinical practice. It is often assessed using the Maternal Antenatal Attachment Scale (MAAS), which covers two dimensions: quality and intensity of attachment. However, studies including the MAAS presented missing or inadequate psychometric properties. Therefore, the current study aimed to investigate the reliability and validity of both the original and the recently introduced brief German version of the MAAS.

**Materials and methods:**

Data from 184 pregnant women from a longitudinal study were used. Women (≥ 18 years old) were recruited between the 18^th^ and 22^nd^ weeks of gestation while waiting for routine prenatal diagnostic appointments. Participants answered the MAAS, together with other questionnaires measuring maternal mental health, self-esteem, and social support. For both versions of the MAAS (19 items vs. 13 items), item characteristics, confirmatory factor analysis, internal consistency, and test-retest reliability were calculated and compared. Moreover, associations between the brief German MAAS and theoretically related constructs were analysed using correlation coefficients.

**Results:**

In this study, item analyses revealed better psychometric properties for the brief German MAAS than for the original MAAS, with a significant reduction in items with inadequate discriminatory power. The internal consistency (*α* ≥ .69) and test-retest reliability (ICC ≥ .62) were acceptable to good for both MAAS versions. With regard to structural validity, factor analysis of the German MAAS presented acceptable to good global model fit indices for the model with correlated factors (GFI > .90; RMSEA ≤ .08; SRMR < .10) in the current sample. In contrast, most global model fit indices of the original MAAS were not acceptable. The construct validity of the German MAAS was demonstrated on the basis of small-to-moderate correlations with a variety of constructs (e.g., measures of depression, anxiety, stress).

**Conclusions:**

According to the present results, the brief German version of the MAAS represents a reliable and valid measurement instrument of MFA for use in clinical practice. Further studies examining possible cut-off values are needed to identify pregnant women with significant attachment difficulties who may benefit from additional support.

## Introduction

During pregnancy, forming a relationship with the unborn child is an essential task, fundamental for adapting to motherhood and ensuring the child’s well-being [1]. According to Condon, maternal-foetal attachment (MFA) refers to the ‘emotional tie or bond which normally develops between the pregnant woman and her unborn infant’ [2, p. 359], manifesting in feelings, thoughts, and behaviours towards the foetus. Condon proposed a hierarchical model of attachment in which the core experience is ‘love’. Based on this, five subjective experiences mediate the relationship between the love experience and overt behaviours: the disposition to know, to be with, to avoid separation or loss, to protect, and to identify and gratify the needs of the object, specifically the foetus [3].

Many studies have investigated the correlations of MFA with a range of sociodemographic (age, education, parity), pregnancy-related (gestational age, planning of pregnancy), and psychological variables (social support, self-esteem, depression, anxiety) [4,5]. For example, higher levels of MFA have been associated with higher levels of pregnancy-related health practices (e.g., abstaining from drugs, obtaining prenatal care) [6,7] and higher levels of social support [2,8,9]. In contrast, lower levels of MFA have been associated with higher levels of perceived stress [10] and greater levels of depressive symptoms during pregnancy and postpartum [11,12]. The results of studies on other aspects of maternal mental health, such as anxiety or eating disorders, have been inconsistent or insufficient [11,13]. In longitudinal studies, the quality of MFA was a significant predictor of postpartum mother-infant bonding [14–17], postpartum maternal sensitivity [18–20], and infant development (e.g., infant temperament, milestone attainment) [21–23].

Given the crucial impact of MFA on adapting to motherhood, child development, and the future mother-child relationship, accurate assessment of MFA is essential for timely identification and support of mothers with attachment difficulties [4]. One of the frequently used self-report measures to assess prenatal attachment is the Maternal Antenatal Attachment Scale (MAAS), developed by Condon [3]. The MAAS is supposed to measure MFA with two independent underlying dimensions: (1) the quality of involvement (11 items, further called *quality*) and (2) the intensity of preoccupation with the foetus (8 items, further called *intensity*). The *quality* subscale refers to the affective experiences in relation to the foetus, such as closeness, tenderness, pleasure in interaction, distress at fantasised loss, and the conceptualisation of the foetus as a ‘real person’. The *intensity* subscale refers to the amount of time spent thinking about, talking to, dreaming about or palpating the foetus and the strength of the accompanying feelings, but not their actual quality. All 19 items have to be rated on a 5-point response scale, with higher values indicating higher MFA. Condon validated the MAAS in a sample of 112 pregnant women and reported good internal consistency (Cronbach’s *α* = .82 for MAAS total). Based on exploratory factor analysis, the 2-factor solution explained 39% of the variance in the MAAS. The MAAS has been translated into different languages, including Dutch [24], Hungarian [25], Italian [26], Spanish [27], Polish [28], and German [29,30].

### Reliability of the MAAS

In line with the original MAAS, the majority of the translated versions showed acceptable to good internal consistency according to the classification of Cohen [31] (*α* = .71 to .87 for MAAS total, *α* = .65 to .80 for *quality*, *α* = .62 to .82 for *intensity*). However, there is a lack of data about the test-retest reliability of the MAAS thus far, which is essential for the accurate assessment of a scale’s structural validity [32]. In his original study, Condon [3] referred to the need for future studies investigating the test-retest reliability of the MAAS.

### Validity of the MAAS

Different aspects of the construct validity of the MAAS have been examined. First, the structural validity of the MAAS has been investigated in several cross-cultural validation studies using confirmatory factor analyses [26–29]. All of them have confirmed two factors similar to the *quality* and *intensity* dimensions proposed by Condon [3] but with different factor loadings of the items compared to the original version. Some authors have proposed substantial item reduction, resulting in brief 11- [28], 12- [27], and 13-item [29] versions of the MAAS. The excluded items (e.g., items 6, 12, 14, 16, 18, and 19) were largely consistent across the studies. The hypothesised orthogonal two-factor model was not supported by the results of the mentioned analyses, which revealed correlated rather than uncorrelated factors. Thus, evidence of the structural validity of the MAAS is controversial, and further factor-analytical examinations of the MAAS are needed. However, it should be taken into account that the use of different brief versions of the MAAS for different countries could prevent international comparability of research results. Second, convergent validity has been underlined by correlations between MAAS scores and alternative measures of prenatal attachment [25,26] and women’s maternal orientations [24]. Third, hypothesised associations between MFA and theoretically related constructs were supported by a series of studies demonstrating that MAAS scores correlated positively with partner relationship satisfaction and social support and negatively with depressive symptoms during pregnancy as well as postpartum [2,26,33,34]. However, at the subscale level, some studies reported only negative correlations between the *quality* subscale of the MAAS and depressive symptoms and no significant correlations with the *intensity* subscale [2,24]. According to van Bussel et al. [24], the intensity of women’s preoccupation with their foetus seems more determined by external factors such as employment.

In summary, recent reviews have underlined the need for future studies analysing the reliability and validity of antenatal attachment scales due to inadequate or missing psychometric properties [32,35]. Considering that a new brief German version of the MAAS [29] contains only 13 items compared to the original version, which has 19 items, analyses of test-retest reliability and construct validity in terms of hypotheses testing for this version are still lacking. Therefore, the current study aimed to address some of the abovementioned gaps. Specifically, the aims were as follows:

1. Analysing and comparing the item characteristics of the original (19-item) and brief (13-item) German MAAS
2. Analysing and comparing the structural validity of the original and brief German MAAS using confirmatory factor analyses
3. Analysing and comparing the reliability of the original and brief German MAAS, including analyses of internal consistency and test-retest reliability
4. Analysing the construct validity of the brief German MAAS in terms of hypotheses testing for the first time by focusing on diverse aspects of maternal mental health and other psychological variables. Based on theoretical assumptions and previous findings [2,5,11,13,24], higher levels of MFA were expected to be associated with lower levels of depression, anxiety, pregnancy-related worries, and perceived stress but not with disordered eating. In addition, it was hypothesised that the associations between the *quality* subscale of the MAAS and variables of maternal mental health would be stronger compared to the *intensity* subscale. Furthermore, higher levels of MFA were expected to be associated with higher levels of self-esteem and social support.

## Methods

### Participants and procedure

The study was conducted in accordance with the Declaration of Helsinki and approved by the Ethics Committee of the Medical Faculty of the University of Leipzig (reference number: 422/17-ek, 14.11.2017). Participants were recruited during routine prenatal diagnostic appointments at the Department of Obstetrics of the University of Leipzig (Germany). Recruitment took place between 4^th^ April 2018 and 23^rd^ December 2019. Eligible women who were pregnant between the 18^th^ and 22^nd^ weeks of gestation and who were at least 18 years old were included. Women with multiple pregnancies and inadequate German reading or writing skills to answer the questionnaires were excluded. Women who agreed to participate in the study received study information and an informed consent sheet. Written informed consent was obtained from all participants before study inclusion. Data for the present analyses were collected via paper-pencil or online questionnaires at two assessment points during pregnancy as part of a larger prospective study: T1 (second trimester: 18^th^-22^nd^ week of gestation) and T2 (third trimester: 33^rd^-37^th^ week of gestation). Nonresponders were contacted by email or postal mail up to two times within three weeks.

### Measures

Sociodemographic variables, including age, nationality, partnership, education, income, parity, and information about previous pregnancies, were obtained through self-generated questions.

The extent of planning and desiring the current pregnancy was measured with two self-generated items on a 5-point response scale. Higher values indicate a greater intensity of planning and desiring the current pregnancy.

MFA during the 14 days prior to the assessment was assessed using a German translation of the Maternal Antenatal Attachment Scale (MAAS) [3,30]. In accordance with the original author of the MAAS, the items were translated using a stepwise forward-backward translation method [30]. The German translation of the MAAS used in this study is provided in S1 File. The MAAS (19 items, total score range 19–95) covers two subscales: quality of attachment (11 items) and intensity of preoccupation with the foetus (8 items). The *quality* sum score ranges from 11 to 55, and the *intensity* sum score ranges from 8 to 40, with higher scores indicating higher MFA. A German validation study [29] proposed a brief version of the MAAS (13 items, total score range 13–65). Despite the item reduction of 6 items, the German version still consists of two factors equivalent to the original *qualit*y (4 items) and *intensity* (9 items) dimensions and has demonstrated acceptable to good internal consistency (*α* = .81 for MAAS total, *α* = .78 for *quality*, *α* = .82 for *intensity*).

Depression severity during the 7 days prior to the assessment was measured using the German version of the Edinburgh Postnatal Depression Scale (EPDS) [36,37]. The EPDS sum score (10 items) ranges from 0 to 30, with higher scores indicating greater severity of depressive symptomatology. A cut-off of ≥ 10 has been established to indicate significantly elevated levels of depression during the second trimester of pregnancy [38]. The German version of the EPDS has demonstrated good reliability [36]. In the present sample, the internal consistency was comparably good (*α* = .84).

Anxiety severity during the 14 days prior to the assessment was measured using the German version of the 7-item Generalised Anxiety Disorder Scale (GAD-7) [39,40]. The GAD-7 sum score (7 items) ranges from 0 to 21, with higher scores indicating greater severity of anxiety symptomatology. A cut-off of ≥ 8 has been established to indicate significantly elevated levels of anxiety [41]. The reliability and validity of the German version of the GAD-7 were confirmed in the general population [39,42]. In the present sample, the internal consistency was good (*α* = .84).

Pregnancy-related worries were assessed using the German version of the Cambridge Worry Scale (CWS) [43,44]. The CWS sum score (17 items) ranges from 0 to 85, with higher scores indicating greater pregnancy-related worries. The German version of the CWS has shown satisfactory psychometric properties [44]. In the present sample, the internal consistency was good (*α* = .85).

Perceived stress during the month prior to the assessment was measured using the German version of the Perceived Stress Scale (PSS-10) [45,46]. The PSS-10 sum score (10 items) ranges from 0 to 40, with higher scores indicating a greater extent of global stress. The reliability and validity of the German version of the PSS-10 were confirmed in the general population [46]. In the present sample, the internal consistency was good (*α* = .85).

Eating disorder psychopathology was measured using the German version of the Eating Disorder Examination-Questionnaire (EDE-Q) [47,48]. The EDE-Q (28 items) covers four subscales: restraint, eating concern, weight concern, and shape concern. The Global Score (mean of the four subscales) ranges from 0 to 6, with higher scores indicating greater severity of eating disorder psychopathology. The German version of the EDE-Q has shown good internal consistency and acceptable validity [48]. In the present sample, all subscales (.80 ≤ *α* ≤ .91) as well as the Global Score demonstrated good internal consistency (*α* = .87).

Self-esteem was measured with the revised German version of the Rosenberg Self-Esteem Scale (RSE) [49,50]. The RSE sum score (10 items) ranges from 0 to 30, with higher scores indicating higher self-esteem. The reliability and validity of the German version of the RSE were confirmed in the general population [51]. In the present sample, the internal consistency was good (*α* = .88).

Social support was measured with the Berlin Social Support Scales (BSSS) [52]. The two subscales perceived social support (8 items) and received social support (11 items) were applied. The mean scores of the subscales range from 0 to 4, with higher scores indicating a greater extent of social support. There is evidence for the reliability and validity of the BSSS [52]. In the present sample, all subscales demonstrated acceptable to good internal consistency (.78 ≤ *α* ≤ .88).

### Statistical analyses

All the statistical analyses were performed using the Statistical Package for Social Sciences, version 25 (IBM® SPSS®), including the software Analysis of Moment Structures, version 25 (IBM® SPSS® Amos). The significance level was set to *α* = .05. Only complete datasets were included (see S2 File for the dataset underlying the results). A-priori power analysis was calculated with the R-package semPower [53]. Power analysis for CFA based on structure equation modelling with a two-factor solution and the specifications RMSEA = .05, *α* = .05, and *β* = .20 resulted in a required sample size of N = 130 (*df* = 151), which was fulfilled in the current study. Analyses were calculated for the original [3] as well as the brief German MAAS [29].

#### Item and scale analyses

At the item level, the mean score (*M*), standard deviation (*SD*), skewness, and kurtosis were calculated as indicators of response distributions on the subscales of the MAAS. Inter-item correlations (*r*) were calculated and expected to be high between items loading on the same factor due to their shared variance and low between items loading on different factors. Item difficulty (P_i_) was computed by referring to the frequency of agreement with the item across the sample [54]. P_i_ ranges from 0 to 100, with high values (> 80.00) indicating that most participants scored high and low values (< 20.00) indicating that most participants scored low on the item. As an indicator of item discriminatory power, corrected item-total correlations for the subscales (*r*_it_^a^) and for the total scale (*r*_it_^b^) were computed and expected to be greater than .40 [55]. Moreover, the mean score (*M*), standard deviation (*SD*), skewness, and kurtosis were calculated as indicators of distributions of the MAAS subscales as well as the total scale. Z-standardisations of skewness (z_skewness_) and kurtosis (z_kurtosis_) were calculated to estimate whether the subscales and the total scale were normally distributed.

#### Structural validity

The structural validity of the MAAS was assessed with confirmatory factor analyses (CFA) based on structure equation modelling using the software AMOS. For each version of the MAAS, the theoretical model proposed by Condon [3] representing *quality* and *intensity* as two uncorrelated latent factors (the correlation coefficient between the latent factors was fixed at 0) was compared with the alternative model representing *quality* and *intensity* as two correlated latent factors using the χ^2^-test. The following fit indices and corresponding cut-off criteria were used to evaluate the global model fit: 1) χ^2^-test, overall testing for the null hypothesis that the model fits the data, 2) Goodness of Fit Index (GFI), with scores > .90 indicating acceptable and scores ≥ .95 indicating good model fit, 3) Root Mean Square Error of Approximation (RMSEA) including the 90% confidence interval, with values ≤ .08 indicating acceptable and scores ≤ .05 indicating good model fit, 4) Comparative Fit Index (CFI) and Tucker-Lewis Index (TLI), with values > .95 indicating acceptable and ≥ .97 indicating good model fit, and 5) Standardised Root Mean Square Residual (SRMR), with values < .10 indicating acceptable and scores < .05 indicating good model fit [56,57]. The global model fit indices were compared at a descriptive level. As local fit indices, standardised factor loadings, their statistical significance, communality as an indicator of explained variance in the items by their designated latent factor, and measurement error variance were considered. Standardised factor loadings > .40 were rated as substantive [58].

#### Reliability

Scale reliability was determined by internal consistency and test-retest analyses. Internal consistency was calculated using Cronbach’s *α*, with *α* ≥ .70 indicating acceptable reliability [58]. To estimate test-retest reliability, all participants were sent the MAAS again at the T2 assessment during the third trimester of pregnancy. Test-retest reliability was determined by calculating a two-way mixed-effects intraclass correlation coefficient (ICC) with a consistency measure of agreement [59]. An ICC < .40 was interpreted as poor, an ICC between .40 and .59 as fair, an ICC between .60 and .74 as good and an ICC ≥ .75 as excellent [60].

#### Hypotheses testing for construct validity

In addition to structural validity, construct validity was also assessed by calculating Pearson’s or Spearman’s rank correlation coefficients between the subscales as well as the total scale of the brief German MAAS and theoretically related constructs, including sociodemographic, pregnancy-related, mental health, and other psychological variables. One-tailed significance tests were performed (except for disordered eating, as no specific direction of effect was hypothesised; therefore, a two-tailed significance test was performed). The sequentially rejective Bonferroni-Holm procedure [61] was used to avoid error rate inflation due to multiple testing by adjusting the rejection criteria for each hypothesis [62]. According to Cohen [31], correlation coefficients of approximately .10 were classified as small, approximately .30 as moderate, and approximately .50 as large effect size.

## Results

### Sample characteristics

Seven hundred and seven women were asked to participate in the study, 452 (63.9%) of whom agreed to participate at the time of recruitment. A total of 222 women returned questionnaires at T1 and T2 (response rate: 49.1%). Of those, 34 women were excluded because they did not receive the MAAS due to a delayed inclusion of the questionnaire in the study material, resulting in a sample of 188 women. For the current analyses, another 4 cases (2.1%) were removed due to missing MAAS data, resulting in a final sample of N = 184 pregnant women.

Table 1 shows the sample characteristics at the T1 assessment. The age of the participants ranged from 21 to 45 years. The majority of women were of German nationality, were in a partnership, and had a high level of school education. Slightly more than half of the women reported a monthly net household income greater than 3.000€. Nearly half of the women had given birth to one or more children before their current pregnancy. Concerning previous pregnancies, 45.0% of women had experienced at least one miscarriage, 12.6% had experienced an abortion, and 4.5% had experienced a stillbirth. Overall, 17.4% of women reported suffering from mental disorders in their lifetime, most often from depression or anxiety disorders. A total of 22.3% of women reported elevated depression symptoms, and 13.0% reported elevated anxiety symptoms during their second trimester of pregnancy.

**Table 1:** Sample characteristics at the T1 assessment.

Sociodemographic characteristics		
Age in years, *M (SD)*		31.60 (4.82)
German nationality, *n* (%)		179 (97.3)
Being in a partnership, *n* (%)		176 (98.3)
Education, *n* (%)		
	Low secondary qualification	5 (2.7)
	High secondary qualification	50 (27.5)
	University entrance qualification	127 (69.8)
Monthly net household income, *n* (%)		
	≤ 1000€	9 (5.0)
	1001–2000€	42 (23.1)
	2001–3000€	39 (21.4)
	3001–4000€	44 (24.2)
	4001–5000€	33 (18.1)
	≥ 5001€	15 (8.2)
**Pregnancy-related characteristics**		
Parity, *n* (%)		
	Nulliparous	93 (50.5)
	Primiparous	60 (32.6)
	> Primiparous	31 (16.9)
Previous pregnancies, *n* (%)		112 (60.9)
	Live birth (≥ 37^th^ week of gestation)	75 (67.6)
	Premature birth (< 37^th^ week of gestation)	11 (9.9)
	Miscarriage	50 (45.0)
	Abortion	14 (12.6)
	Stillbirth	5 (4.5)
**Maternal mental health**		
History of mental disorders, *n* (%)		32 (17.4)
Depression: EPDS ≥10, *n* (%)		41 (22.3)
Anxiety: GAD-7 ≥8, *n* (%)		24 (13.0)

Total sample N = 184 (percentages are calculated from valid cases); *M* = mean; *SD* = standard deviation.

### Item characteristics

Table 2 presents the item characteristics of the original as well as the brief German MAAS. The item means (range 1–5) ranged from 2.05 (item 17) to 4.93 (item 19) for both versions of the MAAS, with low standard deviations, indicating a consistent response pattern among participants with limited variation. For the original MAAS, all items, except for items 5, 8 and 17, were negatively skewed. Skewness ranged from -7.26 (item 19) to 0.95 (item 17). Thirteen items showed positive kurtosis, and six items (1, 2, 4, 5, 6, 7) negative kurtosis. Kurtosis ranged from -0.87 (item 2) to 61.66 (item 19). Negative skewness and positive kurtosis were most pronounced for items 12 and 19. Inter-item correlations for the subscale *quality* ranged from *r* = -.03 to *r* = .59, being particularly small for items 10 *(r* ≤ .12), 12 (*r* ≤ .06), 15 (*r* ≤ .12), 16 (*r* ≤ .15), and 19 (*r* ≤ .13). A large inter-item correlation was found between items 11 and 13 (*r* = .59). Inter-item correlations for the subscale *intensity* ranged from *r* = .07 to *r* = .48, being particularly small for items 14 (*r* ≤ .13), 17 (*r* ≤ .08), and 18 (*r* ≤ .15). The item difficulty scores ranged from P_i_ = 26.22 (item 17) to P_i_ = 98.23 (item 19). No item showed a high, ten items (52.6%) showed a medium, and nine items (47.4%; 3, 9, 11, 12, 13, 15, 16, 18, 19) showed a low item difficulty. The corrected item-total correlations for the subscales ranged from *r*_it_ = .23 (items 16, 19) to *r*_it_ = .59 (item 5), and those for the total scale ranged from *r*_it_ = .18 (item 19) to *r*_it_ = .53 (item 8). Nine items (47.4%; 6, 7, 10, 12, 14, 16, 17, 18, 19) showed a corrected item-subscale correlation of less than .40, indicating inadequate discriminatory power.

**Table 2:** Item characteristics for the original and brief German MAAS factor solutions.

		*M*	*SD*	S	K	P_i_	*r*_it_^a^	*r*_it_^b^
**Original MAAS**								
*Quality*								
3^a^	Positive/negative feelings towards foetus	4.60	0.63	-1.45	1.54	89.95	.53	.41
6^a^	Concept of foetus as ‘person’/’thing’	4.03	1.17	-0.82	-0.67	75.82	.31	.34
7^a^	Well-being of foetus depends on mother	3.57	1.14	-0.63	-0.29	64.27	.30	.36
9^a^	Tender/irritable feelings towards foetus	4.68	0.52	-1.37	0.94	92.12	.49	.38
10^a^	Clear/vague mental picture of foetus	3.46	0.92	-0.53	0.64	61.55	.29	.36
11	Happy/sad feelings towards foetus	4.45	0.67	-0.92	0.18	86.14	.53	.42
12^a^	Absence/desire to hurt or punish foetus	4.88	0.40	-4.05	19.51	97.01	.25	.19
13	Emotionally close/distant feelings towards foetus	4.44	0.65	-1.34	3.84	86.01	.54	.49
15^a^	Anticipate positive/negative first impression of baby	4.81	0.42	-2.03	3.27	95.24	.44	.36
16^a^	Desire to hold baby immediately/later	4.80	0.51	-2.60	5.80	95.11	.23	.22
19	Sad/pleasant feelings towards fantasised foetal loss	4.93	0.39	-7.26	61.66	98.23	.23	.18
*Intensity*								
1^a^	Frequent/infrequent thoughts of foetus	3.60	0.80	-0.18	-0.39	65.08	.56	.43
2	Strong/weak feelings accompanying thoughts of foetus	3.95	0.83	-0.20	-0.87	73.78	.41	.50
4	Strong/weak desire to get information about foetus	3.62	0.98	-0.18	-0.68	65.49	.56	.45
5^a^	Frequent/infrequent picturing of foetus in imagination	2.90	0.99	0.13	-0.74	47.55	.59	.52
8	Frequent/infrequent talking to foetus	2.35	0.99	0.83	0.42	33.83	.52	.53
14	Frequent/infrequent concern regarding mother’s diet	3.63	0.87	-0.86	1.21	65.76	.32	.39
17	Frequent/infrequent dreams about foetus	2.05	0.98	0.95	0.44	26.22	.33	.26
18^a^	Frequent/infrequent palpation of foetus	4.72	0.56	-1.91	2.65	93.07	.25	.25
**German MAAS**								
*Quality*								
3^a^	Positive/negative feelings towards foetus	4.60	0.63	-1.45	1.54	89.95	.60	.38
9^a^	Tender/irritable feelings towards foetus	4.68	0.52	-1.37	0.94	92.12	.53	.31
11	Happy/sad feelings towards foetus	4.45	0.67	-0.92	0.18	86.14	.65	.37
13	Emotionally close/distant feelings towards foetus	4.44	0.65	-1.34	3.84	86.01	.52	.46
*Intensity*								
1^a^	Frequent/infrequent thoughts of foetus	3.60	0.80	-0.18	-0.39	65.08	.54	.48
2	Strong/weak feelings accompanying thoughts of foetus	3.95	0.83	-0.20	-0.87	73.78	.41	.48
4	Strong/weak desire to get information about foetus	3.62	0.98	-0.18	-0.68	65.49	.50	.47
5^a^	Frequent/infrequent picturing of foetus in imagination	2.90	0.99	0.13	-0.74	47.55	.59	.51
7^a^	Well-being of foetus depends on mother	3.57	1.14	-0.63	-0.29	64.27	.31	.36
8	Frequent/infrequent talking to foetus	2.35	0.99	0.83	0.42	33.83	.53	.53
10^a^	Clear/vague mental picture of foetus	3.46	0.92	-0.53	0.64	61.55	.34	.36
17	Frequent/infrequent dreams about foetus	2.05	0.98	0.95	0.44	26.22	.37	.30
18^a^	Frequent/infrequent palpation of foetus	4.72	0.56	-1.91	2.65	93.07	.24	.25

*M* = mean; *SD* = standard deviation; S = skewness; K = kurtosis; P_i_ = item difficulty; *r*_it_^a^ = corrected item-subscale correlation; *r*_it_^b^ = corrected item-total scale correlation.

^a^ Reverse-coded item (higher values indicate higher MFA).

For the brief German MAAS, all items, except for items 5, 8, and 17, were negatively skewed. Skewness ranged from -1.91 (item 18) to 0.95 (item 17). Eight items showed positive kurtosis, and five items (1, 2, 4, 5, 7) showed negative kurtosis. Kurtosis ranged from -0.87 (item 2) to 3.84 (item 13). The differences between the items in terms of skewness and kurtosis were relatively small. Inter-item correlations for the subscale *quality* ranged from *r* = .38 to *r* = .59, being particularly large between items 3 and 11. Inter-item correlations for the subscale *intensity* ranged from *r* = .08 to *r* = .48, being particularly small for items 7 (*r* ≤ .15), 10 (*r* ≤ .09), 17 (*r* ≤ .08), and 18 (*r* ≤ .15). The item difficulty scores ranged from P_i_ = 26.22 (item 17) to P_i_ = 93.07 (item 18). No item showed a high, eight items (61.5%) showed a medium, and five items (38.5%; 3, 9, 11, 13, 18) showed a low item difficulty. The corrected item-total correlations for the subscales ranged from *r*_it_ = .24 (item 18) to *r*_it_ = .65 (item 11), and those for the total scale ranged from *r*_it_ = .25 (item 18) to *r*_it_ = .53 (item 8). Four items (30.8%; 7, 10, 17, 18) showed a corrected item-subscale correlation of less than .40, indicating inadequate discriminatory power. Overall, the item analyses revealed that compared with the original version of the MAAS, the brief German MAAS showed favourable characteristics with regard to item distribution, item difficulty, and corrected item-total correlation.

### Confirmatory factor analyses

Table 3 provides the global model fit indices of the theoretical and alternative model for both versions of the MAAS. For the original MAAS, all values of the global model fit indices associated with the theoretical model with uncorrelated latent factors did not fit the recommended cut-off criteria, except for RMSEA, indicating an acceptable model fit. Furthermore, the χ^2^-difference test revealed a significant difference between the theoretical and alternative model, suggesting that the alternative model with correlated latent factors more accurately reproduced the data. The correlation between the latent factors *quality* and *intensity* was *r* = .402 (covariance = 0.091, *SE* = 0.025, *t* = 3.708). However, all values of the global model fit indices of the alternative model likewise did not fit the recommended cut-off criteria, except for RMSEA and SRMR, both indicating an acceptable model fit.

**Table 3:** Global model fit indices of the theoretical and alternative model, listed for the original and brief German MAAS factor solutions.

		χ^2^ (*df*)	*p* (χ^2^)	GFI	RMSEA (90% CI)	*p* (RMSEA)	CFI	TLI	SRMR
**Original MAAS**									
1.	Theoretical model: Two-factor model with uncorrelated factors	254.823 (152)	< .001	.876	.061 (.047, .074)	.088	.840	.821	.115
2.	Alternative model: Two-factor model with correlated factors	235.793 (151)	< .001	.880	.055 (.041, .069)	.250	.868	.851	.082
	Difference 1 vs. 2	19.030 (1)	< .001						
**German MAAS**									
1.	Theoretical model: Two-factor model with uncorrelated factors	106.686 (65)	.001	.921	.059 (.038, .079)	.217	.912	.894	.112
2.	Alternative model: Two-factor model with correlated factors	92.643 (64)	.011	.929	.049 (.024, .071)	.495	.940	.926	.073
	Difference 1 vs. 2	14.043 (1)	< .001						

GFI = Goodness of Fit Index; RMSEA = Root Mean Square Error of Approximation; CI = confidence interval; CFI = Comparative Fit Index; TLI = Tucker Lewis Index; SRMR = Standardised Root Mean Square Residual.

*p* < .05.

For the brief German MAAS, the global model fit indices of the theoretical model revealed acceptable values for GFI and RMSEA. In contrast, the CFI, TLI, and SRMR did not fit the recommended cut-off criteria. The χ^2^-difference test revealed a significant difference between the theoretical and alternative model, suggesting that the alternative model more accurately reproduced the data. The correlation between the latent factors *quality* and *intensity* was *r* = .351 (covariance = 0.083, *SE* = 0.025, *t* = 3.322). The global model fit indices of the alternative model revealed a good value for RMSEA as well as acceptable values for GFI and SRMR but not for CFI and TLI.

Overall, the alternative model with correlated latent factors showed a more appropriate model fit than did the theoretical model with uncorrelated factors. In particular, the alternative model of the German MAAS displayed acceptable global model fit indices.

Table 4 provides an overview of the local fit indices of the alternative model with correlated latent factors for both versions of the MAAS. For the original MAAS, the standardised factor loadings ranged from .251 (item 16) to .721 (item 11). All factor loadings reached statistical significance (|*t*| > 1.96). Nine items (47.4%; 6, 7, 10, 12, 14, 16, 17, 18, 19) showed standardised factor loadings less than .40, indicating inadequate factor loadings. The explained variance in the individual items by their designated latent factors ranged from 6.3% (item 16) to 52.0% (item 11). The residual variances of the items ranged from 0.119 (item 15) to 1.239 (item 6).

**Table 4:** Local fit indices of the alternative model with correlated latent factors, listed for the original and brief German MAAS factor solutions.

	*λ*	*SE*	*t*	*h* ^2^	Θ
**Original MAAS**					
*Quality*					
3^a^	.689	-	-	.475	0.206
6^a^	.307	0.244	3.716*	.094	1.239
7^a^	.311	0.217	3.759*	.097	1.164
9^a^	.644	0.105	7.401*	.415	0.158
10^a^	.279	0.174	3.387*	.078	0.770
11	.721	0.137	8.102*	.520	0.213
12^a^	.381	0.077	4.571*	.145	0.136
13	.649	0.131	7.450*	.421	0.243
15^a^	.569	0.083	6.640*	.323	0.119
16^a^	.251	0.096	3.057*	.063	0.239
19	.257	0.074	3.117*	.066	0.143
*Intensity*					
1^a^	.653	-	-	.426	0.368
2	.529	0.142	5.863*	.280	0.487
4	.620	0.175	6.657*	.385	0.593
5^a^	.686	0.182	7.147*	.471	0.518
8	.615	0.175	6.613*	.378	0.602
14	.386	0.144	4.444*	.149	0.642
17	.383	0.162	4.416*	.147	0.819
18^a^	.285	0.090	3.354*	.081	0.284
**German MAAS**					
*Quality*					
3^a^	.721	-	-	.520	0.188
9^a^	.601	0.100	6.925*	.361	0.173
11	.790	0.142	8.169*	.623	0.167
13	.601	0.124	6.928*	.361	0.269
*Intensity*					
1^a^	.652	-	-	.425	0.369
2	.524	0.142	5.819*	.275	0.491
4	.598	0.174	6.468*	.357	0.619
5^a^	.681	0.182	7.111*	.464	0.525
7^a^	.340	0.187	3.953*	.115	1.140
8	.626	0.176	6.695*	.391	0.589
10^a^	.359	0.151	4.164*	.129	0.728
17	.408	0.164	4.679*	.167	0.800
18^a^	.282	0.091	3.318*	.080	0.284

*λ =* standardised factor loading; *SE* = standard error of factor loading; *t* = t-value with significance level

*p < .05; *h*^2^ = communality; Θ = measurement error variance.

^a^ Reverse-coded item (higher values indicate higher MFA).

For the brief German MAAS, the standardised factor loadings ranged from .282 (item 18) to .790 (item 11). All factor loadings reached statistical significance (|*t*| > 1.96). Three items (23.1%; 7, 10, 18) showed standardised factor loadings less than .40, indicating inadequate factor loadings. The explained variance in the individual items by their designated latent factors ranged from 8.1% (item 18) to 62.3% (item 11). The residual variances of the items ranged from 0.167 (item 11) to 1.140 (item 7).

### Scale characteristics and reliability

Table 5 presents the scale characteristics and reliability measures of the MAAS. For both versions, the subscale *quality* was negatively and the subscale *intensity* was positively skewed, and both subscales showed positive kurtosis. For the original MAAS, the total scale was negatively skewed, and for the brief German MAAS, it was positively skewed, while both total scales demonstrated negative kurtosis. Z-standardisation of skewness and kurtosis revealed that the subscale *quality* was not normally distributed in either version, whereas the subscale *intensity* and the total subscale were normally distributed in both versions.

**Table 5:** Scale characteristics and reliability of the original and brief German MAAS factor solutions.

	*M*	*SD*	Range	S	K	Cronbach’s *α*		Test-retest^a^
						T1	T2	
**Original MAAS**								
*Quality*	48.66	3.96	35–55	-0.67^b^	0.27^c^	.693	.692	.673
*Intensity*	26.83	4.26	16–38	0.21^b^	0.01^c^	.747	.757	.743
Total scale	75.49	6.97	59–92	-0.04^b^	-0.34^c^	.793	.827	.768
**German MAAS**								
*Quality*	18.17	1.90	11–20	-1.04^b^	0.77^c^	.769	.756	.616
*Intensity*	30.23	4.75	18–43	0.21^b^	0.12^c^	.743	.771	.746
Total scale	48.40	5.67	32–63	0.06^b^	-0.09^c^	.773	.808	.759

*M* = mean; *SD* = standard deviation; S = skewness; K = kurtosis.

^a^ Test-retest interval: *M* = 11.92 weeks (*SD* = 1.77)

^b^ *SE* (standard error) = 0.18

^c^ *SE* = 0.36.

For the original MAAS, the correlations between the *intensity* and *quality* subscale with the total scale were large (*r* = .86 and *r* = .84, *p* < .001, respectively), whereas the correlation between the two subscales was moderate (*r* = .44, *p* < .001). For the German MAAS, the correlations between the *intensity* and *quality* subscale with the total scale were large (*r* = .95 and *r* = .61, *p* < .001, respectively), whereas the correlation between the two subscales was moderate (*r* = .33, *p* < .001).

For the original MAAS, the subscale *quality* demonstrated almost acceptable internal consistency, and the subscale *intensity* demonstrated acceptable internal consistency at each of the two assessment points during pregnancy (T1 and T2). The total scale demonstrated acceptable to good internal consistency at both assessment points. Each of the subscales of the German MAAS demonstrated acceptable internal consistency at T1 and T2. The total scale showed acceptable to good internal consistency at T1 and T2. Deletion of single items within the subscales or the total scale did not improve the internal consistency to a meaningful extent concerning both versions and measurement points.

The test-retest reliability (test-retest interval in weeks: *M* = 11.92, *SD* = 1.77) of each of the subscales of both versions of the MAAS was good and excellent for the total scale of both versions.

### Hypotheses testing for construct validity

Table 6 displays bivariate correlations between the subscales and the total scale of the German MAAS and theoretically related constructs. The *quality* subscale showed significant positive correlations with the extent of desire for the current pregnancy, self-esteem, and perceived social support and significant negative correlations with depression, anxiety, worries during pregnancy, perceived stress, and overall eating disorder psychopathology. All effect sizes were moderate. The *intensity* subscale as well as the total scale showed a significant positive correlation with received social support. The effect size was small. In total, the strongest associations were found between the *quality* subscale and self-esteem, depression, anxiety, and perceived stress.

**Table 6:** Bivariate correlations between the subscales and the total scale of the brief German MAAS and theoretically related constructs.

	German MAAS		
	*Quality*	*Intensity*	Total scale
Age	.118	-.138	-.076
Number of children	.006	.295	.267
School education (low vs. high)	-.006	-.179	-.152
Extent of planning current pregnancy^a^	.137	-.006	.039
Extent of desiring current pregnancy^a^	.282*	.154	.209
Depression (EPDS)	-.385*	.209	.045
Anxiety (GAD-7)	-.341*	.051	-.072
Pregnancy-related worries (CWS)	-.271*	.122	.011
Perceived stress (PSS)	-.318*	.042	-.072
Overall eating disorder psychopathology (EDE-Q Global Score)	-.285*	-.093	-.174
Self-Esteem (RSE)	.426*	.007	.149
Perceived Social Support (BSSS)	.301*	.105	.189
Received Social Support (BSSS)	.127	.225*	.231*

Pearson’s respectively Spearman’s rank correlation coefficient with significance level (one-tailed significance tests except for disordered eating)

*p < .05 (all correlation coefficients with * remained significant after Bonferroni-Holm correction).

^a^ Self-developed item on a 5-point response scale (1 = low, 5 = high), Spearman rank correlation.

## Discussion

The MAAS is a self-report measure that enables a two-dimensional assessment of MFA during pregnancy. The current study compared the psychometric properties of the original version of the MAAS [3] with those of the brief 13-item German version [29], offering significant implications for the clinical adoption of more effective and targeted tools. This study aimed to extend previous work by analysing test-retest reliability and construct validity in terms of hypotheses testing. Analysing the psychometric properties of the MAAS is important for research in the field of MFA as well as for clinical practice, allowing the identification of pregnant women at greater risk for low attachment to their foetus and the development of prevention programmes.

The first aim was to analyse and compare the item characteristics of both versions of the MAAS. Only 2.2% of participants did not answer the items of the MAAS, which indicates a high acceptance rate of the questionnaire among pregnant women. For both versions, most items were negatively skewed and differed between positive and negative kurtosis. However, items of the original MAAS showed more extreme values of skewness and kurtosis than did the German MAAS. Moreover, while both subscales of the original MAAS were characterised by multiple low inter-item correlations, this was only the case for the subscale *intensity* of the German MAAS. Overall, the German version showed more favourable values for item distribution, item difficulty (38.5% vs. 47.4% low difficulty), and discriminatory power (30.8% vs. 47.4% inadequate power) than did the original MAAS in the current sample. These results are in line with previous findings about the low discriminatory power of some items of the original MAAS [27], supporting the use of shorter versions. Indeed, various validation studies have proposed the exclusion of items with insufficient item characteristics, with items 6 (concept of foetus as ‘person’/’thing’), 12 (absence/desire to hurt or punish foetus), 14 (frequent/infrequent concern regarding mother’s diet), 15 (anticipated positive/negative first impression of baby), 16 (desire to hold baby immediately/later), and 19 (sad/pleasant feelings towards fantasised foetal loss) being most frequently mentioned [27–29]. The majority of excluded items belonged to the original *quality* subscale. Despite the overall favourable results for the brief German MAAS, the *intensity* subscale still showed some restrictions at the item level. Items 7, 10, 17, and 18 displayed corrected item-total correlations less than .40, indicating inadequate discriminatory power. Moreover, item 18 showed low item difficulty. As a consequence, other authors excluded item 18 from their adaptation of the MAAS to Spanish (12-item version) [27] or Polish (11-item version) [28].

The second aim was to analyse and compare the structural validity of the original and German MAAS. Two possible conceptual models of the factorial structure of the MAAS were used and compared: a theoretical model proposed by Condon [3], which postulated independence of the two latent factors *quality* and *intensity*, and a more empirically derived alternative model, which postulated interdependence of the two latent factors. For the original MAAS, most global model fit indices deviated from the values suggested as indices of a close fit [56,57] for both the theoretical model with uncorrelated factors and the alternative model with correlated factors. Similarly, for the German MAAS, most indices associated with the theoretical model were not acceptable. In contrast, the alternative model with correlated factors revealed mostly acceptable fit indices. Overall, the alternative model with correlated factors was the one that best reproduced the data for both versions of the MAAS, which is contrary to the theory proposed by Condon [3] postulating independence of the two factors and suggesting a division of four possible attachment styles. According to the present results, a categorisation of pregnant women based on their individual values of the two factors could not be recommended. Moreover, both versions of the MAAS showed significant correlations between each subscale and the total scale (*r* = .61 to *r* = .95) and moderate correlations between the subscales (*r* = .33 to *r* = .44). This is in line with previous studies finding moderate to large correlations between both factors [24,25], supporting the better fit of the alternative model with correlated factors [27]. As this is the first time that the German MAAS was analysed on the basis of its latent factorial structure via confirmatory factor analysis, the results showed that the German version displayed more appropriate structural validity than the original MAAS. However, with regard to the local fit indices of the alternative model of the German MAAS, the standardised factor loadings of items 7 (well-being of foetus depends on mother), 10 (clear/vague mental picture of foetus), and 18 (frequent/infrequent palpation of foetus) were classified as inadequate in the current study. This means that the variance in these items could not be well explained by the variance in the latent factor *intensity*. These results contradict those of Göbel et al. [29], who initially developed the brief German MAAS and found sufficient factor loadings for these items using a sample similar to that used in the current study (e.g., comparable mean age, relationship status, educational level, and household income). At the same time, other validation studies also reported low discriminatory power or factor loadings for the items mentioned, which in some cases led to the exclusion of these items [27,28]. Since the deletion of one of the items did not significantly change the internal consistency, all items of the *intensity* subscale were retained in the present study. Further research is needed to explore whether an even shorter version of the *intensity* subscale of the German MAAS, consisting of 6 rather than 9 items (without items 7, 10 and 18), would maintain comparable psychometric properties. Future studies should also examine the clinical impact of such adaptations in everyday practice. However, the development of different brief versions for different countries could prevent international comparability of study results.

The third aim was to analyse and compare the reliability of both versions of the MAAS. While the subscale *quality* of the original MAAS demonstrated almost acceptable internal consistency, the subscale *quality* of the German MAAS as well as the subscale *intensity* and the total scale of both versions showed acceptable (*α* ≥ .70) to good internal consistency (*α* ≥ .80) at each of the two assessment points during pregnancy (T1 and T2). The internal consistency was lower than that reported in Condon’s original study [3] but comparable to that reported in previous studies, reaching acceptable to good values (.70 ≤ *α* < .90) [24,25,29]. Only the Spanish and Italian validation studies found questionable internal consistency for both subscales (*α* < .70) [26,27]. Although the internal consistency of the German MAAS was higher than that of the original MAAS in the present study (especially for the subscale *quality*), the test-retest reliability was comparable (ICC ≥ .60) for both versions, which was analysed for the first time.

The last aim was to analyse the construct validity of the brief German MAAS in terms of hypotheses testing for the first time. Overall, the significant correlations observed between the German MAAS and the analysed constructs as well as the corresponding directions aligned mostly with the assumptions of the present study. With regard to maternal mental health, higher levels of MFA were associated with lower levels of depression and anxiety. However, this was only demonstrated for the subscale *quality*. Previous studies have shown ambivalent results, finding negative correlations between depression and anxiety with both subscales as well as the total scale of the MAAS [2,25] and between depression and anxiety and only the *quality* subscale [24,34]. Differences seem to emerge in relation to the measurement instrument used. For example, Condon and Corkindale [2] reported that depression measured with the EPDS [37] was less strongly correlated with the MAAS than was depression measured with the Hospital Anxiety and Depression Scale—depression subscale (HADS-D) [63]. This could be because the EPDS includes items not covered in the HADS-D, e.g., items targeting anxiety, self-harm or self-blame. In contrast, the HADS-D mainly focuses on quality of mood, especially the capacity for enjoyment. Additionally, the review of Göbel et al. [13] reported mixed results for the association between the *intensity* subscale as well as the total scale of the MAAS and different anxiety measures across studies but stable negative correlations with the *quality* subscale of the MAAS. Furthermore, higher levels of MFA were associated with lower levels of pregnancy-related worries and perceived stress. In line with previous studies [10,64], these correlations were found only for the *quality* subscale of the MAAS. In contrast to current assumptions, higher levels of MFA *quality* were associated with lower levels of disordered eating. A Chinese study reported a significant negative correlation between the *quality* subscale of the MAAS and postpartum disordered eating (bulimic symptoms) [65]. However, as disordered eating has rarely been studied in relation to MFA [11], further studies are needed to identify broader trends. Overall, not all aspects of the MFA concept seemed to be affected by maternal mental health status. While the *quality* of attachment appeared to be determined by internal states such as mood, the *intensity* of women’s preoccupation with the foetus seemed to be more determined by external factors such as life events or the presence of other children [2]. The present findings underscore the value of differentiating the total attachment score into its qualitative and quantitative dimensions, as suggested by previous literature [24]. Furthermore, they confirmed the need for mental health screening in pregnant women to provide additional support, particularly for high-risk populations, in promoting MFA.

With regard to other psychological variables, higher levels of MFA were associated with higher levels of self-esteem, which is in line with a study of an adolescent sample [64]. In contrast, the study by Koniak-Griffin [66] revealed no correlation between MFA and self-esteem in adolescents. The differences in the results could be due to the use of different measures of MFA and self-esteem. In line with the review of McNamara et al. [11], higher levels of MFA were associated with higher levels of social support. In detail, perceived social support correlated positively with the *quality* subscale, and received social support correlated positively with the *intensity* subscale as well as the total scale of the MAAS. The current results extend previous work in differentiating the effects of perceived and received social support, as recommended by the meta-analysis of Yarcheski et al. [5]. Perceived social support appeared to be more important for MFA than actually received social support [2,67]. Social support may act as a protective factor when individuals are faced with stressful and challenging situations such as pregnancy. Nevertheless, as the results are cross-sectional, further research investigating the potential changes in social support during pregnancy in relation to the need for social support among pregnant women would be interesting.

Furthermore, higher levels of MFA *quality* were associated with a greater desire to become pregnant. This finding is in line with other studies showing greater MFA in women with wanted pregnancies [25,68], demonstrating the close relationship between attitudes towards pregnancy and MFA. Further research is recommended to explore the factors behind unplanned and unwanted pregnancies and their associations with MFA. Additionally, no associations with sociodemographic factors were found, which is in line with a previous meta-analysis [5] showing that demographic factors did not contribute significantly to MFA.

In summary, the brief German version of the MAAS developed by Göbel et al. [29] showed acceptable to good reliability and structural validity in a sample of pregnant German women and appeared to be more favourable than the original version developed by Condon [3]. Furthermore, the present study provided evidence in support of the construct validity of the German MAAS for the first time, based on associations with a variety of psychological variables. Therefore, the use of the brief German MAAS for German-speaking mothers is recommended in the context of clinical practice. To identify women with prenatal attachment difficulties who need additional support, further research is needed to investigate cut-off values for low MFA. Moreover, since most associations with mental health variables depended on the subscale used, the results emphasise the importance of distinguishing between the two attachment dimensions *quality* and *intensity*.

### Limitations

The results must be interpreted in light of several limitations. First, the recruitment of participants from a university hospital in a larger German city and the requirement of fluent German language skills may have introduced selection bias, potentially resulting in a sample that is not entirely representative of the German population. The sociodemographic characteristics of the participants in the present study were rather uniform. Most women had a high level of education, had a medium to high income, were living with a partner, and reported having a German nationality. These aspects might limit the generalisability of the results to other pregnant populations. Thus, the psychometric properties of the brief German MAAS should be further explored in more diverse samples in future studies. Moreover, a relatively high number of eligible pregnant women did not participate in the current study. It can be assumed that the lack of time and interest in the study might be possible reasons. In addition, recruiting women immediately before their routine prenatal diagnostic appointments might not have been ideal, as many women were more or less in a tense and anxious mood because they were not yet sure whether their baby was healthy. However, high attrition rates are not uncommon in longitudinal studies with a sample of pregnant women. In a review of studies investigating ante- and postpartum depression in women, one-quarter of the studies reported attrition rates between 37.3% and 49.9% [69]. Second, the MAAS is a self-report instrument measuring subjective MFA in expectant mothers. Future studies may examine its consistency with various objective assessment approaches, such as observation of maternal behaviour. Third, the statistical power was limited due to the sample size and multiple comparisons. Nevertheless, Bonferroni-Holm correction was used to avoid alpha-level inflation due to multiple testing, which was an unattended problem of previous studies. Fourth, a causal interpretation of the identified associations is not possible, as the construct validity was determined on the basis of cross-sectional data.

## Conclusions

Despite these limitations, the MAAS allows a time- and cost-effective assessment of MFA during pregnancy and has been validated among pregnant women. In the present study, the brief German version of the MAAS showed acceptable to good psychometric properties. Therefore, the German MAAS represents a reliable and valid instrument with significant implications for both clinical practice and public health policies in Germany, particularly for practitioners in obstetric, paediatric and primary care. Integrating the MAAS as a routine screening tool in prenatal care and national health initiatives could help to detect attachment difficulties earlier, offer tailored interventions, and promote preventive programs aimed at enhancing MFA. Such efforts could improve the long-term mental health of mothers as well as the developmental outcomes of children. The positive impact of psychoeducational interventions in prenatal settings, as demonstrated by Diotaiuti et al. [70], underscores the importance of combining MFA assessments with targeted interventions to improve maternal psychosocial well-being during pregnancy. In the context of scientific research, the use of the complete 19-item version of the MAAS is recommended to maintain international comparability of study results. In addition, an evaluation can be carried out for the brief 13-item German version. Based on the data collected, the psychometric properties should be reviewed and critically discussed.

## Supporting information

S1 File German translation of the MAAS by Wittich et al. (2009) [30]. (DOCX)

S2 File Dataset underlying the results described in the manuscript. (XLSX)
