# Supplementary material for: Reliability and validity of the original and brief German version of the Maternal Antenatal Attachment Scale (MAAS): Longitudinal study findings
Source: PLoS One. 2024 Dec 31;19(12):e0316374. doi: 10.1371/journal.pone.0316374 (PMC11687779; doi:10.1371/journal.pone.0316374)
Supplement: S1 File — (DOCX) [file pone.0316374.s001.docx]

**Diese Fragen beziehen sich auf Ihre Gedanken und Gefühle über das heranwachsende Baby. Bitte kreuzen Sie zur Beantwortung jeder Frage nur ein Kästchen an. Es gibt keine richtigen oder falschen Antworten.**

1. In den vergangenen zwei Wochen habe ich über

das Baby in meinem Bauch nachgedacht oder war damit beschäftigt.

1. In den vergangenen zwei Wochen, als ich über das Baby in meinem Bauch sprach oder nachdachte, waren meine Gefühle:
2. In den vergangenen zwei Wochen waren meine Gefühle für das Baby in meinem Bauch:
3. In den vergangenen zwei Wochen hatte ich den Wunsch, etwas über das heranwachsende Baby zu lesen oder Informationen zu bekommen. Dieser Wunsch war:
4. In den vergangenen zwei Wochen habe ich versucht mir vorzustellen, wie das heranwachsende Baby in meinen Bauch eigentlich aussieht.
5. In den vergangenen zwei Wochen habe ich mir das heranwachsende Baby vorgestellt als:
6. In den vergangenen zwei Wochen habe ich gespürt, dass das Wohlergehen des Babies in meinem Bauch von mir abhängig ist.
7. In den vergangenen zwei Wochen habe ich bemerkt, dass ich mit dem Baby gesprochen habe, als ich allein war.
8. In den vergangenen zwei Wochen, als ich über das Baby in meinem Bauch nachdachte (oder mit ihm sprach), waren meine Gedanken:
9. Meine Vorstellung, wie das Baby in meinem Bauch gegenwärtig aussieht, ist:

- beinahe die ganze Zeit
- sehr häufig
- häufig
- gelegentlich
- gar nicht
- sehr schwach oder nicht vorhanden.
- eher schwach.
- mal schwächer, mal stärker.
- eher stark.
- sehr stark.
- sehr positiv.
- eher positiv.
- mal positiv, mal negativ.
- eher negativ.
- sehr negativ.
- sehr schwach oder nicht vorhanden.
- eher schwach.
- mal schwächer, mal stärker.
- eher stark.
- sehr stark.
- beinahe die ganze Zeit
- sehr häufig
- häufig
- gelegentlich
- gar nicht
- eine richtige kleine Person mit besonderen Eigenschaften.
- ein Baby wie jedes andere Baby auch.
- einen Menschen.
- etwas Lebendiges.
- etwas, was noch nicht richtig am Leben ist.
- völlig
- erheblich
- mäßig
- etwas
- gar nicht
- gar nicht
- gelegentlich
- häufig
- sehr häufig
- beinahe die ganze Zeit, als ich allein war
- immer zärtlich und liebend.
- eher zärtlich und liebend.
- eine Mischung aus Zärtlichkeit und Gereiztheit.
- eher etwas gereizt.
- häufig gereizt.
- sehr klar.
- eher klar.
- eher vage.
- sehr vage.
- Ich habe überhaupt keine Vorstellung.

1. In den vergangenen zwei Wochen, wenn ich über das Baby in meinem Bauch nachdachte, waren meine Gefühle gegenüber dem Baby:
2. Einige schwangere Frauen werden wegen des Babies in ihrem Bauch manchmal so gereizt, dass sie daran denken, es zu verletzen oder zu bestrafen:
3. In den vergangenen zwei Wochen fühlte ich mich gegenüber meinem Baby:
4. In den vergangenen zwei Wochen habe ich darauf geachtet, was ich esse, damit mein Baby gut ernährt wird.
5. Wenn ich mein Baby das erste Mal nach der Geburt sehen werde, werde ich vermutlich:
6. Wenn mein Baby zur Welt gekommen ist, möchte ich das Baby in den Armen halten.
7. In den vergangenen zwei Wochen habe ich von der Schwangerschaft oder dem Baby geträumt.
8. In den vergangenen zwei Wochen habe ich bemerkt, dass ich mit meiner Hand über die Stelle an meinem Bauch taste oder reibe, wo sich das Baby befindet.
9. Wenn ich mein Baby jetzt ohne irgendwelche eigenen Schmerzen oder Verletzungen (wegen einer Fehlgeburt oder einem Unfall) verlieren würde, würde ich mich:

- sehr traurig.
- eher traurig.
- eine Mischung aus Glücksgefühlen und Traurigkeit.
- eher glücklich.
- sehr glücklich.
- Ich kann mir nicht vorstellen, dass ich mich jemals so fühlen würde.
- Ich kann mir vorstellen, dass ich mich mitunter so fühlen könnte, habe mich aber noch nie so gefühlt.
- Ich habe mich ein- oder zweimal so gefühlt.
- Ich habe mich gelegentlich so gefühlt.
- Ich habe mich oft so gefühlt.
- sehr distanziert.
- eher distanziert.
- nicht besonders verbunden.
- eher verbunden.
- sehr verbunden.
- gar nicht
- ein oder zweimal beim Essen
- gelegentlich beim Essen
- relativ oft beim Essen
- jedes Mal beim Essen
- intensive Zuneigung fühlen.
- größtenteils Zuneigung fühlen.
- Ablehnung gegenüber ein oder zwei Eigenschaften des Babys fühlen.
- Ablehnung gegenüber ziemlich vielen Eigenschaften des Babys fühlen.
- hauptsächlich Ablehnung fühlen.
- sofort
- nachdem es in ein Tuch gewickelt wurde
- nachdem es gewaschen wurde
- nach ein paar Stunden, wenn sich alles beruhigt hat
- am nächsten Tag
- gar nicht
- gelegentlich
- häufig
- sehr häufig
- beinahe jede Nacht
- mehrmals am Tag
- mindestens einmal am Tag
- gelegentlich
- nur einmal
- gar nicht
- sehr zufrieden fühlen.
- eher zufrieden fühlen.
- weder traurig noch zufrieden fühlen oder gemischte Gefühle haben.
- eher traurig fühlen.
- sehr traurig fühlen.
